# The Plant Cell Wall: A Complex and Dynamic Structure As Revealed by the Responses of Genes under Stress Conditions

**DOI:** 10.3389/fpls.2016.00984

**Published:** 2016-08-10

**Authors:** Kelly Houston, Matthew R. Tucker, Jamil Chowdhury, Neil Shirley, Alan Little

**Affiliations:** ^1^Cell and Molecular Sciences, The James Hutton InstituteDundee, UK; ^2^Australian Research Council Centre of Excellence in Plant Cell Walls and School of Agriculture, Food and Wine, Waite Research Institute, The University of AdelaideGlen Osmond, SA, Australia

**Keywords:** cell walls, biotic, abiotic, stress, gene expression

## Abstract

The plant cell wall has a diversity of functions. It provides a structural framework to support plant growth and acts as the first line of defense when the plant encounters pathogens. The cell wall must also retain some flexibility, such that when subjected to developmental, biotic, or abiotic stimuli it can be rapidly remodeled in response. Genes encoding enzymes capable of synthesizing or hydrolyzing components of the plant cell wall show differential expression when subjected to different stresses, suggesting they may facilitate stress tolerance through changes in cell wall composition. In this review we summarize recent genetic and transcriptomic data from the literature supporting a role for specific cell wall-related genes in stress responses, in both dicot and monocot systems. These studies highlight that the molecular signatures of cell wall modification are often complex and dynamic, with multiple genes appearing to respond to a given stimulus. Despite this, comparisons between publically available datasets indicate that in many instances cell wall-related genes respond similarly to different pathogens and abiotic stresses, even across the monocot-dicot boundary. We propose that the emerging picture of cell wall remodeling during stress is one that utilizes a common toolkit of cell wall-related genes, multiple modifications to cell wall structure, and a defined set of stress-responsive transcription factors that regulate them.

## Introduction

The plant cell wall is a complex structure that fulfills a diverse array of functions throughout the plant lifecycle. In addition to maintaining structural integrity by resisting internal hydrostatic pressures, the cell wall provides flexibility to support cell division, a biochemical scaffold that enables differentiation, and a pathological and environmental barrier that defends against stress (Scheller and Ulvskov, 2010; Hamann, 2012; Tucker and Koltunow, 2014). The cell wall hosts a wide range receptors, pores and channels that regulate molecular movement and responses to local and long-range elicitors including hormones, sugars, proteins, and RNAs. Consistent with a role in many processes, plant cell wall structure is incredibly varied, not only between plant species but also between tissue types. In general, two wall types surrounding plant cells are often referred to as the primary wall and secondary wall. A dynamic primary wall is established in young cells during division and acts to provide flexibility and basic structural support, protecting the cell, and mediating cell-cell interactions. The thicker and more durable secondary wall lies between the primary wall and plasma membrane, and is deposited at a later stage when the cell has stopped growing and dividing. The secondary wall is seen as a crucial adaptation that allows terrestrial plants to withstand and facilitate upright growth.

Typical components of the cell wall include cellulose, non-cellulosic, and pectic polysaccharides, proteins, phenolic compounds, and water. The major components (>90%) are polysaccharides, the structure, and biosynthesis of which have been extensively reviewed in recent times (Atmodjo et al., 2013; Pauly et al., 2013; Rennie and Scheller, 2014; Kumar et al., 2016). In brief, cellulose is a water insoluble carbohydrate found in both primary and secondary cell walls whose fibrous structure enables the maintenance of structural integrity. Pectins, which are arguably the most complex and heterogeneous of the cell wall polysaccharides, exist predominantly in the primary cell wall and have roles in expansion, strength, porosity, adhesion, and intercellular signaling. Other abundant non-cellulosic polysaccharides include xyloglucan, β-1,3:1,4-glucan, xylan, mannan, and callose, which fulfill various roles in mechanical support, reserve storage and development. In contrast to cellulose, the pectic and non-cellulosic polysaccharides can be further distinguished by sugar substitutions and side chains that are attached to the polysaccharide backbone during biosynthesis (Scheller and Ulvskov, 2010). These substituents influence solubility, viscosity, and interactions with other polysaccharides and proteins within the cell wall.

The function of different cell wall components and how they interact with exogenous stimuli such as pathogens and environmental stress has been of interest for many years, particularly in the search for mechanisms by which pathogen-resistance, stress-tolerance and improved crop yields might be achieved. Recent reviews have explored how abiotic cues modify cellulose biosynthesis (Wang et al., 2016), how expansins and peroxidases influence wall stiffness during stress (Tenhaken, 2014), and how modifications in non-cellulosic polysaccharides such as xyloglucan accompany stress responses (Le Gall et al., 2015). Only recently have studies begun to consider a broader view of how different stresses might induce similar changes in transcript abundance (Coolen et al., 2016). The question of whether cell wall components, cell wall-related gene families, or indeed individual orthologous cell wall genes, respond in the same way to different stresses in different species has not been addressed in detail, but might provide new broad-specificity targets for the modification of stress responses. Some of the answers may be buried within publically available transcriptome datasets from monocot and dicot species, which detail global transcriptional responses to pathogens such as bacteria, fungi, oomycetes, insects and nematodes, and abiotic stresses such as drought, cold and heat. These datasets provide a resource to identify carbohydrate-related gene families that encode proteins with similar functional domains Carbohydrate-Active enZYmes (CAZy) Database; (Lombard et al., 2014), and to determine whether specific families such as glycosyltransferases (GTs), glycosylhydrolases (GHs), and other carbohydrate-modifying enzymes might play key roles in cell wall synthesis and modification during stress. Therefore, following the first two sections of this review, where we have considered new and historical findings regarding the role of cell wall-related polymers and genes during biotic and abiotic stress, we have revisited multiple transcriptional datasets to summarize the response of cell wall and carbohydrate-related genes and families upon stress, emphasizing the remarkable level of conservation in responses induced by different stress types.

## Biotic stress and the plant cell wall

In the co-evolutionary battle ground between plants and microbes over millions of years, plants have evolved a multi-layered defense system in which the cell wall serves multiple purposes. The plant cell wall may serve as preformed or passive structural barrier as well as an induced or active defense barrier. Microbes have to circumvent the cell wall and other preformed barriers to establish the desired pathogenic relationship with host plants. This requires appropriate host recognition strategies and the development of suitable infection structures and/or chemical weapons (Zentmyer, 1961; Turrà et al., 2015). Failure to evolve appropriate strategies to breach the host wall and other preformed structures results in the microbes becoming non-pathogens and non-adapted pathogens.

The host plant can also use the cell wall as an active defense barrier for those microbes that have evolved a mechanism to overcome the preformed barriers. During infection, oligosaccharide elicitors are released from the host plant's cell wall (damage-associated molecular patterns, DAMPs) or from the pathogen cell wall (pathogen-associated molecular patterns, PAMPs) as a result of degradation (Boller and Felix, 2009). Plants perceive these elicitors through plasma membrane immune receptors that trigger signaling cascades to activate numerous defense responses called DAMP or PAMP triggered immunity (DTI or PTI; Jones and Dangl, 2006). One common DTI or PTI associated defense response is reinforcement of the cell wall in order to create more resistance to physical pressure and/or enzymatic hydrolysis generated by the pathogens (Boller and Felix, 2009; Ringli, 2010; Malinovsky et al., 2014). Depending on the interaction type, the cell wall reinforcement process may occur in several different ways, including rearrangement and cross-linking pre-existing cell wall materials, incorporation of easily cross-linked polymerized materials to the existing cell wall and local deposition of cell wall materials at the infection sites (Moerschbacher and Mendgen, 2012).

### Papillae composition and biotrophic pathogens

The local deposition of cell wall materials, also known as papillae, is an early defense response commonly formed against infection by a number of biotrophic, hemi-biotrophic, and bacterial pathogens (Bellincampi et al., 2014). The tiny micrometer scale structure, formed at the site of infection, is often big enough to halt fungal penetration. In several non-host and host species resistance is achieved at pre-invasion stage due to formation of papillae at the infection sites. However, the exact role of papillae is not well understood. They may act as a physical barrier that effectively halts pathogen penetration or slows down the penetration process so that other defense mechanisms can be activated ahead of time (Stone and Clarke, 1992; Huckelhoven, 2005). They may also function as a chemical barrier that accommodates a variety of chemical weapons like antimicrobial toxins, phytoalexins, and defensins, which are needed to directly attack the pathogens or inhibit the cell wall degrading enzymes produced by pathogens (Albersheim et al., 2011).

It has been hypothesized that papillae-mediated penetration resistance is the ability of a host genotype to develop an effective papillae with the correct composition and at the right time (Aist and Israel, 1977; Inoue et al., 1994). Therefore, understanding papillae composition and factors involving the development of effective papillae have been a focus for many researchers. In the last three decades, studies have attempted to identify the components of papillae formed against different biotrophic pathogens in different crops. While some of the physiochemical changes that occur during papillae-mediated cell wall reinforcement are now well described, many aspects are poorly understood. For example, there has been a lot of research focused on papillary callose accumulation and lignification due to the availability of fluorescent stains for callose and the inherent autofluorescence of lignin compounds, while the potential roles of many other cell wall components remain unknown.

The recent development of cell wall-specific antibodies, carbohydrate binding modules, and small molecule stains provide a new opportunity to capture information about the three dimensional changes of polysaccharides at the infected sites of the cell wall. Chowdhury et al. (2014) utilized these new tools to show that the major polysaccharides found in barley papillae induced in response to the fungal pathogen *Blumeria graminis* f.sp. *hordei* (*Bgh*) are callose, arabinoxylan and cellulose. Effective papillae that are successful in preventing the penetration attempts of *Bgh* contain significantly higher concentrations of these polysaccharides compared to ineffective papillae. The papillae are layered with an inner core consisting of callose and arabinoxylan, and an outer layer containing arabinoxylan and cellulose. The association of arabinoxylan and cellulose with penetration resistance opens new targets for the improvement of papillae composition and generation of lines with improved disease resistance. Previous studies described candidate gene expression profiles during papillae formation and discussed their likely functions in defense (Bhuiyan et al., 2009). However, other that the implication of the *glucan synthase-like* (*GSL*) gene family in the synthesis of papillary callose (Jacobs et al., 2003) the genes involved in the synthesis of the remaining papillae polysaccharides are not yet characterized.

### Necrotrophic pathogens

The interaction between the plant and necrotrophic pathogens occurs at a higher level than observed with biotrophic pathogens. Whilst it is still an aim of the plant cell to prevent pathogen entry, pathogens with a necrotrophic phase of their life cycle have evolved an armory of cell wall degrading enzymes designed to degrade the plant cell wall, along with a range of virulence factors or toxins in order to kill the host cells and release the nutrients within, rather than taking it by stealth (van Kan, 2006). The plants generally respond to the necrotrophic pathogens in a stronger, but similar manner to biotrophic pathogens by reinforcing the cell wall at the point of attack, and modifying the cell wall to be more recalcitrant to enzymatic digestion. This process is often hijacked by the pathogen to its benefit, forcing the plant to alter its cell wall to be more digestible (Hok et al., 2010). Given the widespread damage that can be caused by the toxins, a large wounding response could also be expected due to a loss of cell wall integrity (Ferrari et al., 2013). A majority of the necrotrophic pathogens infiltrate plant tissues through stomata and open wounds spreading between the cell junctions.

Nafisi et al. (2015) reviewed the role of the cell wall in the plant:necrotroph interaction focussing on the downstream phytohormone signaling. The recognition of PAMP signals leads to activation of signaling cascades which interconnect with auxin, cytokinin, brassinosteroids and abscisic acid to activate expression of defense related genes. The susceptibility of the cell wall to degradation and downstream production of PAMPs is therefore important to the resistance of a plant to pathogens. This is demonstrated by the impact of pectin methylesterification in plant-pathogen interactions. Lionetti et al. (2012) reviewed the role of pectin methylesterases in response to a number of plant pathogens, including necrotrophs, highlighting that the de-esterification of pectin affects the susceptibility of the cell wall to fungal cell wall degrading enzymes. A meta-analysis of pectin modifying enzymes in Arabidopsis was performed, however, the glycosyltransferase families implicated in the synthesis of pectin were not included.

### Plant-parasitic nematodes

Cell wall remodeling during parasitic nematode infestation of plant roots is likely to be an essential component for successful completion of the nematode life cycle (reviewed in Bohlmann and Sobczak, 2014). Parasitic nematodes must penetrate, migrate and establish feeding structures (syncytia or giant cells), all of which require some interaction with the root cell walls. Early studies in cyst nematodes investigated the role of cell wall degrading enzymes that are secreted in order to penetrate and migrate to the optimal feeding site (reviewed in Deubert and Rohde, 1971), and recent studies confirm that a cocktail of enzymes such as cellulases, 1,3-β-glucanases and pectin lyases, generally associated with plant pathogenesis, are conserved between different parasitic nematode species (Rai et al., 2015). More recently the focus has shifted to the response of the plant cell wall as it is remodeled to accommodate the formation of a feeding site (reviewed in Wieczorek, 2015) and the differences observed in a susceptible and resistant interaction. Several studies have shown specific changes in wall polysaccharides, such as pectin, during infection (Davies and Urwin, 2012), and hypothesized that cell wall components such as 1,3-β-glucan or 1,3:1,4-β-glucan may influence the solute flow between the nematode and host (Hofmann et al., 2010; Aditya et al., 2015).

### Herbivorous insects

The plant response to attack by herbivorous insects is regulated heavily by the wounding response generated by the recognition of DAMPs (Boller and Felix, 2009). The mechanical damage caused by the insect feeding may be reduced by thickening of the cell wall, however, resistance is more likely to take the form of chemical defense such as phenolics, alkaloids, terpenoids, or glucosinolates (reviewed in van Dam, 2009). Direct targeting of the chitin or other carbohydrate structures present in the insect feeding structures and midgut by plant glycosyl hydrolases or lectins plays a prominent role in herbivore defense by interfering with the nutritional uptake of the pathogen (reviewed in Vandenborre et al., 2011).

### Consequences of modified cell wall composition on pathogenesis

Both gain- and loss-of-function transgenic and genetic approaches have been utilized to examine the effects of altered wall composition on plant disease resistance, several of which are summarized in Table 1. These studies indicate that modified cell wall composition can indeed lead to increased or decreased disease resistance phenotypes in host plants, depending on the target polysaccharide and whether the cell-wall related gene was over-expressed or mutated. In many cases the cell-wall related target genes were identified through transcriptomic methods after the application of a specific biotic stress (see references in Tables 1, 2). However, it is also important to note that a number of these studies were aimed toward improving digestibility of forage crops to render lignocellulose less recalcitrant for bioprocessing, and there is some concern that plants with increased digestibility due to altered cell wall properties might be more susceptible to pests and disease. Evidence from studies of transgenic lines with altered transcript levels of candidate genes involved in cellulose, non-cellulosic polysaccharides, and lignin biosynthetic pathways suggest this may not be the case. For example, reduction of cellulose biosynthesis by means of genetics or chemicals leads to cell wall integrity compensatory effects resulting in increased lignification and enhanced disease resistance (Hamann, 2012).

**Table 1:** **Plant:biotic stress resistance phenotypes with altered cell wall composition**.

**Gene name**	**Mutation/over-expression**	**Host species**	**Observed wall phenotype**	**Pathogen species**	**Phenotype**	**References**
*CesA3*	Mutation	*A. thaliana*	Reduced cellulose in primary wall, lignification, enhanced defense signaling	*Golovinomyces cichoracearum*	R	Ellis and Turner, 2001; Caño-Delgado et al., 2003
				*Golovinomyces orontii*		
				*Oidium lycopersicum*		
*CesA4(irx5)*	Mutation	*A. thaliana*	Defective secondary cell wall, enhanced defense signaling	*Ralstonia solanacearum*	R	Hernández-Blanco et al., 2007
*CesA7(irx3)*				*Plectosphaerella cucumerina*		
*CesA8(irx1)*				*Pseudomonas syringae*		
				*Botrytis cinerea*		
*WAT1*	Mutation	*A. thaliana*	Defective secondary cell wall, enhanced defense signaling	*Xanthomonas campestris*	R	Denancé et al., 2013
				*Verticillium dahlia*		
				*V. alboatrum*		
				*R. solanacearum*		
				*P. cucumerina*		
*Gsl5 (PMR4)*	Mutation	*A. thaliana*	Reduced callose accumulation in papillae, hyperactivation of SA responsive genes	*Sphaerotheca fusca*	R	Jacobs et al., 2003; Nishimura et al., 2003
				*G. orontii*		
				*G. cichoracearum*		
				*Peronospora parasitica*		
*Gsl5 (PMR4)*	Over-expression	*A. thaliana*	Increased callose accumulation in papillae	*G. cichoracearum*	R	Ellinger et al., 2013
*CslF6*	Mutation	*O. sativa*	Reduced mixed-linkage glucan in primary wall, activation of marker PR genes and SA responsive genes	*X. oryzae* pv. *oryzae*	R	Vega-Sánchez et al., 2012
G-proteins	Mutation	*A. thaliana*	Reduced xylose content in the wall	*P. cucumerina*	R	Delgado-Cerezo et al., 2012
*PMR6*	Mutation	*A. thaliana*	Enhanced pectin accumulation	*G. cichoracearum*	R	Vogel et al., 2002
*PMR5*	Mutation	*A. thaliana*	Enhanced pectin accumulation	*G. cichoracearum*	R	Vogel et al., 2004
				*G. orontii*		
*RWA2*	Mutation	*A. thaliana*	Decreased levels of acetylated cell wall polymers	*B. cinerea*	R	Manabe et al., 2011
*PMEI-1*	Over-expression	*A. thaliana*	Increased pectin methyl-esterification activity	*B. cinerea*	R	Vega-Sánchez et al., 2012
*PAL, CCoAOMT, COMT, CAD*	RNAi	*T. monococcum*	Putatively reduced lignification in papillae and epidermal cell wall	*Blumeria graminis* f.sp. *tritici*	S	Bhuiyan et al., 2009
*AtBG_papp*	Mutation	*A. thaliana*	Increased callose deposition at PD	*H. schachtii*	R	Hofmann et al., 2010
*PLL18*	Mutation	*A. thaliana*	Modified pectin content?	*H. schachtii*	R	Wieczorek et al., 2014
				*Meloidogyne incognita*		
*PLL19*	Mutation	*A. thaliana*	Modified pectin content?	*H. schachtii*		Wieczorek et al., 2014
				*M. incognita*		
*AtCel6*	Over-expression	*G. max*	Modified cellulose content?	*H. glycines*	R	Woo et al., 2014
				*M. incognita*		
*GmCel7*	Suppression	*G. max*	Modified cellulose content?	*H. glycines*	R	Woo et al., 2014

*Details of a selection of studies which have identified specific genes that have been linked to changes in the plant cell wall phenotype when the plant is subjected to biotic stress*.

**Table 2:** **Plant:stress systems collated from PLEXdb for meta-analysis**.

**PLEXdb experiment number**	**GSE experiment number**	**Host species**	**Stress type**	**Stress name**	**References**
AT19		*A. thaliana*	Abiotic	Cold	Craigon et al., 2004
			Abiotic	Drought	
AT31	GSE12856	*A. thaliana*	Biotic (biotrophic fungus)	*Blumeria graminis* f.sp. *hordei* (barley powdery mildew)	Jensen et al., 2008
AT49	GSE5525	*A. thaliana*	Biotic (necrotrophic fungus)	*Alternaria brassicola* (black spot)	De Vos et al., 2005
			Biotic (insect)	*Pieris rapae* (small cabbage worm)	
			Biotic (insect)	*Frankliniella occidentalis* (western flower thrips)	
			Biotic (insect)	*Myzus persicae* (green peach aphid)	
AT51	GSE5684	*A. thaliana*	Biotic (necrotrophic fungus)	*Botrytis cinerea* (gray mould)	
AT52	GSE5685	*A. thaliana*	Biotic (bacteria)	*Pseudomonas syringae*	
AT54	GSE5731	*A. thaliana*	Abiotic	UV-A	
			Abiotic	UV-B	
			Abiotic	Visible light	
			Biotic (oomycete)	*Phytophthora parasitica*	
AT59		*A. thaliana*	Biotic (biotrophic)	*Golovinomyces orontii* (powdery mildew)	
AT63	GSE6516	*A. thaliana*	Biotic (insect)	*Bemisia tabaci* (silverleaf whitefly)	Kempema et al., 2007
AT100	GSE18329	*A. thaliana*	Biotic (oomycete)	*Hyaloperonospora parasitica* (downy mildew)	Bhattarai et al., 2010
AT115	GSE14332	*A. thaliana*	Abiotic	Wounding	
AT123	GSE22671	*A. thaliana*	Abiotic	Dark	González-Pérez et al., 2011
			Abiotic	High light	
AT138	GSE37553	*A. thaliana*	Biotic (nematode)	*Meloidogyne incognita* (root-knot nematode)	
BB9	GSE33407	*H. vulgare*	Biotic (necrotrophic fungus)	*Fusarium graminearium* (head blight)	Bodd et al., 2006
BB61	GSE33401	*H. vulgare*	Biotic (hemibiotrophic fungus)	*Cochliobolus sativus* (spot blotch)	Millett et al., 2009
			Biotic (biotrophic fungus)	*Puccinia hordei* (brown rust)	
BB63	GSE14521	*H. vulgare*	Abiotic	Boron	Öz et al., 2009
BB65	GSE10332	*H. vulgare*	Abiotic	Cold	Svensson et al., 2006
BB71	GSE12584	*H. vulgare*	Biotic (insect)	*Rhopalosiphum padi* (bird cherry-oat aphid)	Delp et al., 2009
BB74	GSE17238	*H. vulgare*	Biotic (biotrophic fungus)	*Puccinia graminis* (stem rust)	McGrann et al., 2009
BB79	GSE8618	*H. vulgare*	Biotic (bacteria)	*Pseudomonas aeruginosa*	
BB81	GSE10329	*H. vulgare*	Abiotic	Freezing	
BB83	GSE15295	*H. vulgare*	Abiotic	Mercury	
BB89	GSE17669	*H. vulgare*	Abiotic	Drought (seed)	Abebe et al., 2010
BB92	GSE43906	*H. vulgare*	Biotic (bacteria)	*Pseudomonas syringae*	Colebrook et al., 2012
BB102	GSE23896	*H. vulgare*	Abiotic	Heat	Mangelsen et al., 2011

## Abiotic stress

Another type of external stimulus that can influence the plant cell wall is abiotic stress. This type of stress includes a range of factors such as extreme temperature, drought, flooding, salinity, atmospheric pollutants, and heavy metal contaminants. Often a plant is subjected to multiple abiotic stresses simultaneously which can make it challenging to identify which stress is responsible for the observed response. Various changes in plant cell wall composition under a spectrum of abiotic stresses have been studied and recently reviewed in detail. Wang et al. (2016) discussed the effect of four types of abiotic stress; salt stress, water availability, light conditions and temperature, on one aspect of the plant cell wall, cellulose. Among the genes discussed in detail are members of the *CesA* gene family, which are known to synthesize cellulose, and others that have been previously identified as interacting with the *CesA*s. Le Gall et al. (2015) provide an overview of the influence drought, heat, cold, salt, heavy metal, light, and air pollutant stresses can have on the main components of the plant cell wall in both monocots and dicots. In contrast Tenhaken (2014) focused on the effect of reactive oxygen species (ROS), which are a plant stress response, on components of the plant cell wall such as *XTH* and expansins. Members of the expansin and *XTH* gene families often show differential expression under abiotic stress conditions, and therefore increased presence of ROS, which leads to a potential pause in growth. In this section of the current review (and in Table 3) we provide a brief summary of studies that reveal insight into the transcriptional dynamics of cell wall genes during abiotic stress, before focussing on studies in monocots and dicots that have directly attributed the effect of a cell wall-related gene or gene family to altered abiotic stress responses.

**Table 3:** **Plant: abiotic stress response phenotype with altered cell wall composition**.

**Gene name**	**Mutation/over expression**	**Species**	**Abiotic stress**	**Observed wall related phenotype**	**References**
**UP- /DOWN-REGULATION**					
*Cinnamoyl-CoA reductase 1, Cinnamoyl-CoA reductase 2*	Up regulation	*Z. mays*	Water deficient	Increased lignification in roots	Fan et al., 2006
*CslF6*	Down regulation	*O. sativa*	Flooding (submearged)	Reduced mixed-linkage glucan in primary wall	Kimpara et al., 2008
*CslF6*	Mutation	*H. vulgare*	Susceptable to chilling	Reduced mixed-linkage glucan in primary wall	Taketa et al., 2012
*CTL1*	Mutation	*A. thaliana*	Increased sensitivity to heat, salt, and drought stress	Enlargement of cells and incomplete cell wall Cell walls are cellulose-deficient	Zhong et al., 2002; Mouille et al., 2003; Kwon et al., 2007
*Myb41*	Overexpression	*A. thaliana*	Increased sensitivity to desiccation	Altered cell expansion	Cominelli et al., 2008
*CesA8 (irx1)*	Mutations	*A. thaliana*	Increased tolerance to drought and salinity stress	Thinner secondary cell wall due to less Cellulose, leading to collapse of xylans.	Turner and Somerville, 1997; Chen et al., 2005
*ZmEXPA1, ZmEXPA3, ZmEXPA5, ZmEXPB1, ZmEXPB2, and ZmXET1*	Up regulation	*Z. mays*	Increased salinity	cell enlargement and root swelling	Li et al., 2014

*Details of a selection of studies which have identified specific genes that have been linked to changes in the plant cell wall phenotype when the plant is subjected to abiotic stress*.

### Global profiling of abiotic stress responses

Transcriptional changes that accompany various abiotic stresses have been discussed in considerable detail (reviewed in Santos et al., 2011; Gehan et al., 2015), but remarkably few have considered these changes in the context of specific cell wall-related genes. A detailed analysis of the genetic responses to drought in specific organs of the barley spike was carried out by Abebe et al. (2010). Transcriptional profiles of the awn, seed, lemma, and palea were compared between plants that were drought-stressed due to not receiving water for 4 days during grain filling, and control plants. For all tissues except the seed, multiple cell wall related genes were found to be differentially regulated between the control and the drought stressed plants. Genes encoding members of the cellulose synthase (GT2, CesA), UDP-xylosyltransferase, glycosyl hydrolase family 1 (GH1), endo-beta-1,4-glucanase (GH9), and xyloglucan endotransglycosylase (GH16, XTH/XET) families were among the cell wall-related genes observed to be downregulated in drought conditions. An additional *XET* was upregulated under drought conditions as well as a putative xylanase inhibitor, endo-1,3-beta-glucosidase and beta-D-glucan exohydrolase. Similar studies in Arabidopsis identified over 500 genes that respond to drought, cold, and salinity stress (Seki et al., 2002), including several members of the extensin, pectinesterase, and XTH/XET families that were downregulated. Wang et al. (2013) showed that in the case of salinity alone, over 140 cell wall-related genes respond to salt stress, and sometimes in a differential manner between Arabidopsis ecotypes. As previously identified by other authors under drought conditions (Wu and Cosgrove, 2000; Moore et al., 2008), depending on which tissue is being observed, the plant cell wall is either loosened or tightened in order to maintain growth. This illustrates the complexity of the cell wall response to abiotic stresses.

Plants can experience abiotic stress to differing levels of severity, therefore many studies include several levels of stress treatment to capture how this affects the response. Harb et al. (2010) assessed the effect of progressive drought and moderate drought on plant growth using a range of biochemical and physiological assays, and changes in gene expression were monitored using a microarray experiment. Under moderate drought conditions plant growth was significantly decreased both in terms of biomass accumulation and leaf expansion, as was stomatal conductance. In these conditions several genes encoding cell wall expansins were upregulated; however under progressive drought conditions cell wall expansins were downregulated. Expansins are proteins that have previously been shown to loosen and modify the plant cell wall during growth and adaptation to stress by modifying the cellulose and non-cellulosic components of the cell wall (Cosgrove, 2005). In a similar approach, Mangelsen et al. (2011) exposed young barley caryopses to 0.5, 3, and 6 h of heat stress and used microarrays to identify differentially expressed genes. Down-regulated genes associated with the cell wall were statistically over-represented, particularly after 3 and 6 h of exposure to heat stress, which the authors described as the primary heat response and heat stress adaptation phases respectively. This set included genes functionally annotated as pectate lyases, polygalacturonases, and pectin esterases.

Other approaches have compared transcriptomic data from stress-sensitive and tolerant cultivars. Cal et al. (2013) compared transcriptome data for the leaf elongation zone (LEZ) from two rice cultivars, Moroberekan, which is drought tolerant, and IR64, which is drought sensitive, in water deficit conditions. This tissue was chosen as the changes in expansion in the LEZ are often one of the earlier responses to water deficit (Cutler et al., 1980). These transcriptomic datasets identified a set of genes that showed a >2-fold expression change in both cultivars, including 27 cell-wall related genes, the majority of which were down-regulated in the drought tolerant cultivar Moroberekan. The down-regulated list included genes involved in secondary cell wall production including cinnamoyl-CoA reductase, ferulate-5-hydroxylase, laccase, and apoplastic class III peroxidases. Genes encoding arabinogalactan proteins and extensins, involved in cell wall signaling, and structure, XTH/XETs and GTs, including two *CesA* genes, were differentially expressed between the two cultivars. The two genes found to be upregulated in Moroberekan are members of glycosyl hydrolase family GH28, encoding polygalacturonases. In a similar study, Zheng et al. (2010) compared genome-wide gene expression data for Han21 and Ye478, a drought tolerant and a drought intolerant maize line respectively, under drought stress conditions. A total of 15 probe sets that encode putative cell wall related genes were differentially expressed between the two lines. These included probes annotated as cellulose synthase subunits, endo-1,3-β-glucosidase precursors and COBRA-like 3 protein precursors.

As mentioned previously in this review, plants are often exposed to multiple stresses concurrently, including those that are both abiotic and biotic, and the effects of these are not necessarily simply additive (Puranik et al., 2012; Coolen et al., 2016). Therefore, it is important, despite the obvious complexity of such experiments, to study the effect of multiple simultaneous stresses on plants. *AtRALFL8* was observed to be upregulated in roots of 10 day old plants when dual stresses of nematode infection and water deficit were applied (Atkinson et al., 2013). Subsequent microarray analysis revealed that under these conditions *AtRALFL8* is highly co-expressed with pectinases, known for their capacity to contribute to cell wall remodeling, having previously been demonstrated to have a role in several stress responses including nematode infection (Pelloux et al., 2007; An et al., 2008). Coolen et al. (2016) exposed Arabidopsis plants to single and double combinations of drought stress, fungal *Botrytis cinerea* infection, and herbivorous *Pieris rapae* infection. A total of 41 cell-wall related genes including *PECTIN METHYLESTERASE 3* (*PME3*), *EXPANSIN A6* (*EXPA6*), *XTH10*, and *XTH32* responded to at least one stress, while 12 genes including *CELLULOSE SYNTHASE LIKE G2* (*CSLG2*), *ARABINOGALACTAN PROTEIN 2* (*AGP2*), *BETA GLUCOSIDASE 46* (*BGLU46*), a 1,3-β-glucanase, and *EXPANSIN A8* (*EXPA8*) responded in the same way to all three. This indicates that common transcriptional responses, and possibly downstream effects on cell wall composition, are employed in the response to distinct stresses.

### Genetic and transgenic evidence supporting a role for cell wall genes in abiotic stress responses

Similar to that described for biotic stresses, mutant lines have been a valuable resource in terms of understanding how cell wall-related genes can alleviate or enhance responses to abiotic stress. In Arabidopsis, *AtCesA8*/*IRX1* contributes to the synthesis of the secondary cell wall and influenced plant tolerance to drought and osmotic stress (Chen et al., 2005). Mutant alleles of *AtCesA8, leaf wilting 2-1* (*lew2-1*), and *lew2-2*, showed higher tolerance to osmotic stresses, induced by exposure to NaCl and mannitol, and drought stress compared to wild type plants. Other components of the plant cell wall influence plant tolerance to chilling or frost. For example, Taketa et al. (2012) screened for sodium-azide induced barley mutants that were susceptible to chilling. Of the 11 lines identified, 2 were found to lack (1,3:1,4)-β-glucan and contain mutations in *HvCslF6*, a cell-wall related gene that has previously been implicated in the synthesis of (1,3:1,4)-β-glucan (Burton et al., 2006). While these lines did not contain mutations that would produce a premature stop codon, one mutation resided in close proximity to the conserved *HvCslF6* glycosyltransferase (GT2) catalytic motif. Taketa et al. (2012) hypothesized that the increased sensitivity of vegetative tissues to chilling in lines containing mutations in *HvCslF6* could be due to thinner cell walls, since (1,3:1,4)-β-glucan is usually a major component of this structure in the grasses. This hypothesis was strengthened by the work of Cu et al. (2016) who observed thinner cell walls in *CslF6* knock down lines generated by RNAi when compared to wild type using both a Calcofluor staining method and immunocytological staining with the BG1 (1,3:1,4)-β-glucan specific antibody. Interestingly, (1,3:1,4)-β-glucan content in cereal grain appears to be particularly sensitive to environmental conditions, although it is unclear whether this variation depends only on modified *HvCslF6* function. Swanston et al. (1997) and Wallwork et al. (1998) identified considerable differences in barley grain (1,3:1,4)-β-glucan content depending on the field site or temperature during grain maturation. Similarly, in several wheat varieties grown in different heat and drought conditions, a decrease in grain (1,3:1,4)-β-glucan content was observed in lines grown under stressed conditions (Rakszegi et al., 2014). Conversely an increase in grain arabinoxylan content was reported under the same conditions, which possibly promoted a decrease in (1,3:1,4)-β-glucan. The genetic basis for these variable responses to abiotic stress has yet to be revealed.

Transgenic plants have also been used to validate the role of candidate genes potentially involved in cell wall production/modification and abiotic stress. XET/XTH enzymes are typically thought to have a role in cell wall loosening and therefore cell expansion (Rose et al., 2002). Transgenic Arabidopsis lines expressing a *XTH* from *Capsicum annuum* show abnormal leaf phenotypes including irregular cell patterns in transverse sections and curled leaves (Cho et al., 2006). In addition, transgenic Arabidopsis and tomato (Choi et al., 2011) lines expressing the *Capsicum XTH* showed increased salt tolerance and had longer roots than control plants lacking the transgene, suggesting a role for wall flexibility in alleviating stress responses. In Maize root tissues, multiple cell wall related genes were found to be differentially expressed under salt stress (Li et al., 2014) including *ZmXET1. ZmXET1* is thought to be involved in cell wall extension as it is capable of hydrolyzing and re-joining xyloglucan molecules (Fry et al., 1992). Other genes identified by Li et al. (2014) as being up-regulated when plants were subjected to increased salinity, and therefore possibly involved in mediating resistance against salinity related toxicity, were the expansins *ZmEXPA1, ZmEXPA3, ZmEXPA5, ZmEXPB1, ZmEXPB2*. The expression of these cell wall-related genes may be under epigenetic control, since increased expression of the *ZmHATB* and *ZmGCN5* histone acetyltransferase genes was increased after salt stress, and was accompanied by increased histone H3K9 and H4K5 acetylation. In a separate study by Liu et al. (2014) the overexpression of *OsBURP16* was found to increase the amount of polygalacturonase (PG), an enzyme which hydrolyses pectin, and change the composition of the plant cell wall. Consequently, rice plants overexpressing *OsBURP16* showed less tolerance to drought (quantified as survival after depriving 2 week-old plants of water), with wild type plants showing 42% survival rate compared to <10% for *OsBURP16* overexpression lines. Measuring levels of H_2_O_2_, an indicator of stress, showed that *OsBURP16* overexpression lines were also more susceptible to salt stress than wild type plants.

### Revealing the cell wall stress response network

As we learn more about the networks of genes regulating plant cell wall synthesis and hydrolysis, it is possible that by association, more genes will be identified that are involved in stress response. Recently a detailed study that used both *in vitro* and *in vivo* methods to comprehensively characterize the network of genes regulating secondary cell wall synthesis in Arabidopsis, also highlighted how one part of this network was influenced by abiotic stress (Taylor-Teeples et al., 2015). The authors described the xylem regulatory network, and how changes in both salinity and iron can introduce perturbations, which in turn produced phenotypic changes in the secondary cell wall.

There are a great deal of data available from previous studies on abiotic stress that detail the global response of gene transcription, and in some few cases an assessment of changes in cell wall phenotypes as a response. Similar datasets are available for biotic stresses applied to diverse species and tissues. It is evident from the genetic studies reviewed above that similar gene family members (e.g., the XET/XTH, expansin, and pectin modifier families) are often involved in the response to different stresses. However, the inherent complexity of the wall and the large number of genes involved in its synthesis and modification means that many details remain unclear in regards to the genetic and biochemical basis for cell wall responses to stress. Despite the obvious difficulties involved in comparing experiments between different species, stresses and tissues, in the final section of this review we have revisited public transcriptome datasets to highlight broad similarities between different stress types, and consider whether more attention might be focussed on putative cell wall-related genes that have been overlooked previously.

## Publically available datasets highlight complex transcriptome level responses to abiotic and biotic stress

The previous sections of this review summarized research conducted on various aspects of cell wall reinforcement and modification during pathogen infection and abiotic stress. Cell wall reinforcement in the form of papillae is a relatively common mechanism that determines the outcomes of infection. However, given the diversity of biotic stresses that can be exposed to a plant, any commonalities in papillae formation would likely be accompanied by a range of distinct cell wall-related defense responses. The same might be expected for different abiotic stresses such as extreme temperature, salinity and flooding. In terms of the overlap between biotic and abiotic stresses, a recent study in Arabidopsis showed that ~25% of the cell-wall related transcripts that responded to fungal infection, herbivory, or drought showed a similar response in each treatment (Coolen et al., 2016). Whilst it is currently not possible to perform a detailed review of all cell wall changes induced during the response to a range of different biotic and abiotic stresses, it is possible to perform a meta-analysis using publically available transcript expression data of plant-pathogen and plant-stress interactions in order to highlight overlaps in the responses of the cell wall machinery.

Gene expression data is available from the Plant Expression Database (PLEXdb; Dash et al., 2012), including many microarray datasets from Arabidopsis and barley that detail changes in transcript abundance following exposure to various abiotic or biotic stresses (Table 2). An additional resource of considerable use is the Carbohydrate-Active enZYmes (CAZy) Database (Lombard et al., 2014), which describes families of structurally-related enzymes that hydrolyse, modify or create glycosidic bonds. Using this information, putative *Arabidopsis* CAZy genes present on the Affymetrix 22K ATH1 genome array were selected. Protein family (Pfam) domains associated with the CAZy database annotations were used to identify barley carbohydrate-related genes present on the 22K Barley1 GeneChip. The normalized transcript levels for each carbohydrate-related gene from *Arabidopsis* and barley were compared following each stress (relative to untreated controls) within each experiment and represented as a log(2)-fold induction. As might be expected from the previous sections of this review, many cell wall genes showed pronounced responses to the different stresses.

**Figure 1:**
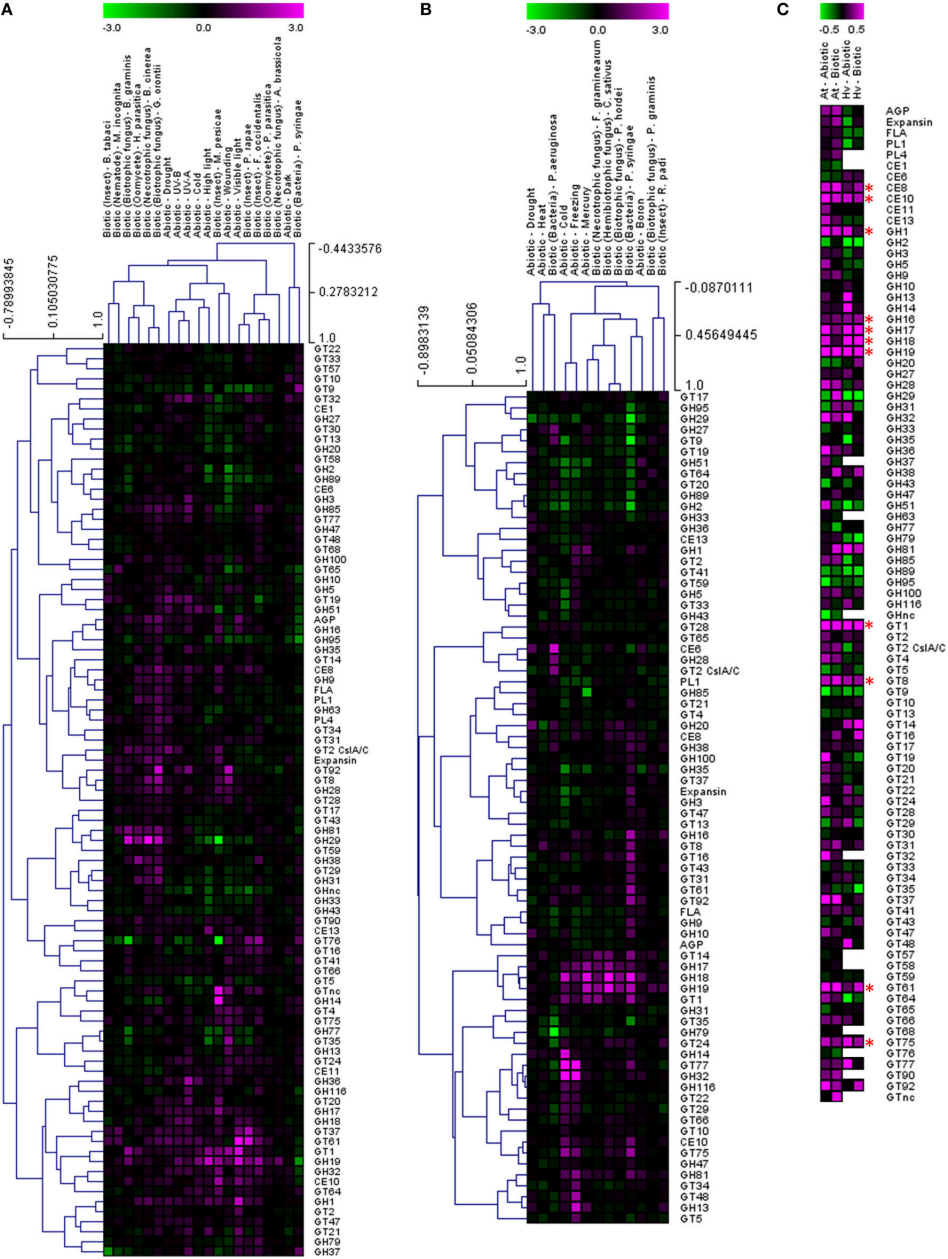
**Analysis of cell wall-related transcripts following abiotic and biotic stresses in Arabidopsis (A) and barley (B)**. Transcript abundance was determined through meta-analysis of microarray datasets collected from the Plant Expression Database (PLEXdb; Dash et al., 2012) using the experiments listed in Table 2. Values show the average log(2)-fold induction for representatives of each CAZy gene family present on the Arabidopsis Affymetrix 22K ATH1 genome array and the 22K Barley1 genechip. Hierarchical clustering was performed based on the Pearson correlation coefficients across each dataset and CAZy family. Trends conserved in response to the stresses between Arabidopsis and barley are observed in **(C)** which shows the average fold induction for each gene family for all abiotic and all biotic stresses in Arabidopsis and barley. Asterisks indicate gene families for which expression is upregulated by both abiotic and biotic stresses in Arabidopsis and barley.

**Figure 2:**
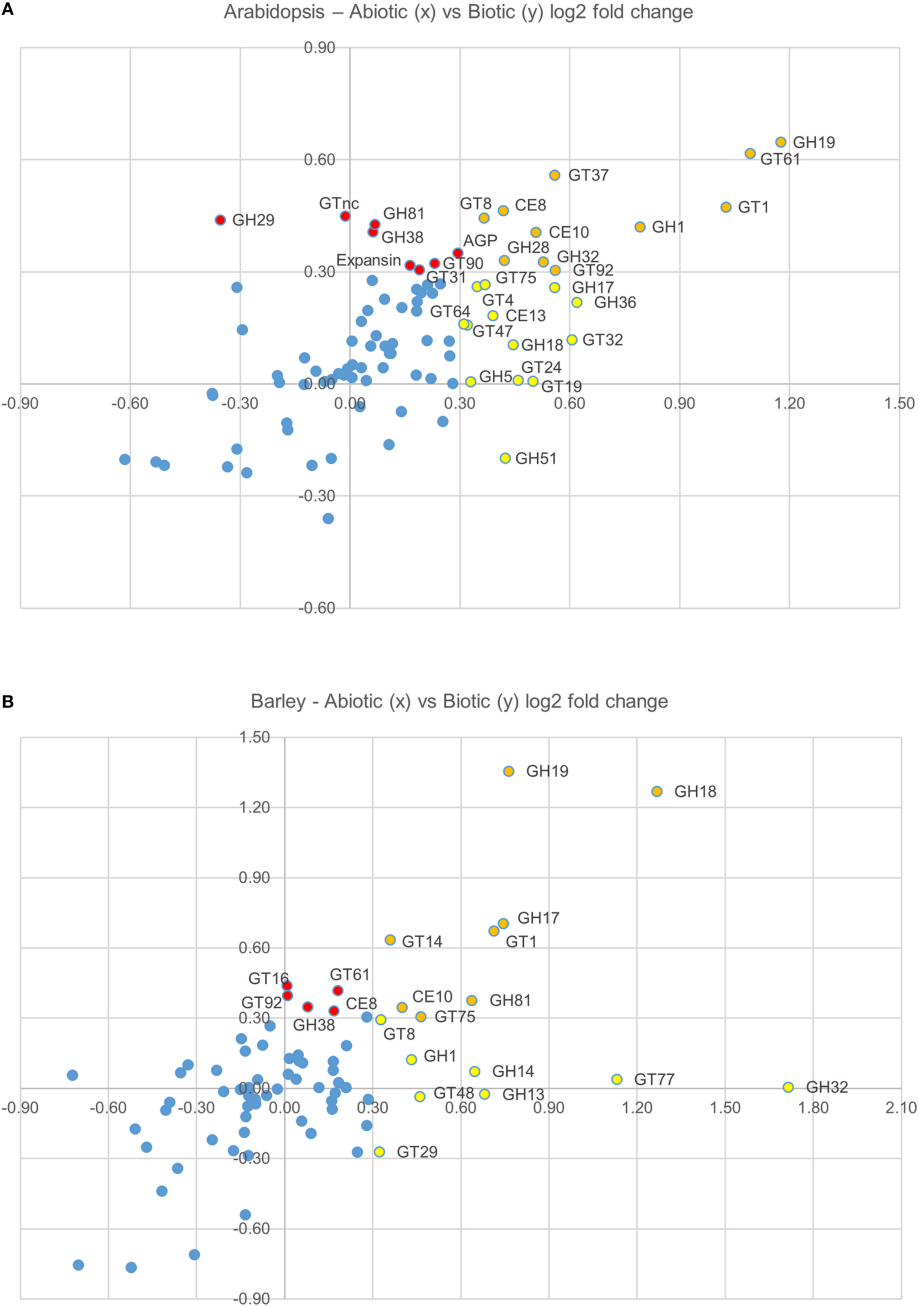
**Graphical representation the average log(2)-fold induction for each gene family (presented in Figure 1C), which shows the average abiotic (*x* axis) and average biotic (*y* axis) stress response in Arabidopsis (A) and barley (B)**. CAZy families that are upregulated in response to abiotic stresses, but not biotic stresses are colored red, CAZy families that are upregulated in response to biotic stresses, but not abiotic stresses are colored yellow, and CAZy families that are upregulated in response to both abiotic and biotic stresses are colored orange.

In order to test whether these responses might be more generally conserved on a CAZy gene family level, the average fold induction observed across all family members was calculated and analyzed using the TIGR Multiexperiment Viewer (MeV). Hierarchical clustering was used to arrange gene families according to similarity in pattern of gene expression (Figures 1A,B) (Eisen et al., 1998). Figures 1A,B clearly demonstrate that most CAZy gene families are upregulated in response to an abiotic or biotic stress in *Arabidopsis* and barley. Although not all CAZy families contain members that act on the same substrate, and the likelihood of all specialized family members responding in the same way is remote, this approach was targeted toward providing a simple means of identifying key carbohydrate-related activities that are shared between different stresses. Similar behaviors of well-characterized and poorly-characterized CAZy families may provide useful insight into novel stress-related cell wall and carbohydrate-related changes. To identify trends conserved in response to the stresses between *Arabidopsis* and barley, the fold induction for each gene family was averaged for all abiotic and all biotic stresses and presented in Figure 1C. The comparative responses of these genes families to abiotic and biotic stress are shown in Figure 2 in both species.

Two different clustering methods have been considered. Within each species the gene families can be clustered based on the correlation of their transcript profile across each experiment. Second, the experiment datasets can also be clustered based on the correlation of the gene family transcript profiles. It is interesting to note that the abiotic stresses generally form a cluster together, as do the biotic stresses, even though there is a vast difference in the type of stress within each cluster (Figures 1A,B). The dogma that the standard cell wall defense response is primarily driven by callose and the *glucan synthase-like* genes of the GT48 CAZy family is hard to support, given the large number of gene families that appear to be upregulated across most stresses. Even though the individual genes induced within each experiment are different, the clustering of CAZy families across the experiments suggests that there is a similar defense response mounted irrespective of the exact stress type. We can see examples of biotrophic fungi inducing similar responses to necrotrophic fungi, drought stress inducing similar responses to cold stress and even examples across different tissues with nematodes in roots compared to whitefly infested leaves.

### Knowledge-based identification of carbohydrate-related families that respond to biotic and abiotic stress

Clustering the response of CAZy families identifies activities that appear to be generally upregulated across most of the experiments, and therefore cell wall or carbohydrate components may be altered similarly during the interaction. Figure 1C depicts the average fold induction for each CAZy gene family across all of the Arabidopsis abiotic stresses, the Arabidopsis biotic stresses, the barley abiotic stresses and the barley biotic stresses. There are examples of CAZy families that only appear to be up-regulated (on average) in Arabidopsis, including arabinogalactan proteins (AGP), expansins, fasciclin-like arabinogalactan proteins (FLA), pectin lyase (PL1), pectin acetylesterase (CE13), glycosyl hydrolases (GH9, GH85), and a range of glycosyl transferases (GT4, GT20, GT21, GT47, and GT64). The number of CAZy families found to be higher on average in barley include xylan acetyl esterase (CE6) and glycosyl transferase (GT14). Given the differences between barley and Arabidopsis cell walls, with barley walls containing more arabinoxylan and (1,3:1,4)-β-glucan and Arabidopsis walls containing more pectin and xyloglucan (Burton et al., 2010), it is not unexpected to see a greater representation of pectin modifying enzymes in the Arabidopsis dataset.

Twelve CAZy gene families are up-regulated (on average) across abiotic and biotic stresses in Arabidopsis and barley. These include polysaccharide degrading and modifying enzymes such as pectin methylesterase (CE8), carbohydrate esterase (CE10), and glycosyl hydrolases (GH1, GH17, GH18, and GH19) which target a range of polysaccharides and oligosaccharides containing 1,3-β-glucan and chitin. Some of these CAZy families have already been implicated in stress-responses as pathogenesis-related (PR) proteins. GH17 genes have been classed as 1,3-β-glucan degrading PR-2 proteins (Leubner-Metzger and Meins, 1999), while GH18 and GH19 represent five of the 17 families of plant PR proteins (Minic, 2008). GH1 family members have been implicated in the activation of defense compounds via the removal of a β-glucoside (Poulton, 1990; Duroux et al., 1998). Pectin methylesterases modify the esterification status of pectin in the wall affecting the susceptibility of the cell wall barrier to fungal and bacterial CWDEs (Collmer and Keen, 1986). De-esterification of pectin also influences the porosity of the plasmodesmata, which can alter the spread of signaling molecules during the defense response (Chen et al., 2000).

The role of the CAZy glycosyltransferase families during the defense response is less characterized than the hydrolytic enzymes. Remarkably, there are five CAZy GT families that are upregulated on average across abiotic and biotic stresses in *Arabidopsis* and barley, including GT1, GT8, GT61, GT75, and GT92. The GT1 family includes a large number of genes with a wide range of putative functions including UDP-glucuronosyltransferase activity. By transferring sugars to a wide range of secondary metabolites, UGTs increase the stability and solubility of aglycones and therefore modify their bioactivity and effectiveness as regulators of the defense response (Lim and Bowles, 2004; Langlois-Meurinne et al., 2005). The GT8 family catalyse the transfer of diverse sugars (Glc, Gal, GlcNAC, GalA) onto lipo-oligosaccharide, protein, inositol, oligosaccharide or polysaccharide acceptors using nucleotide sugar substrates (Yin et al., 2011). Members of the family have been implicated in several different functions including the synthesis of pectins and xylan, and the raffinose family of oligosaccharides that play a role in stress response (Kim et al., 2008). To date GT61, GT75, and GT92 families have not been reported to be involved in the plant defense response. GT61 family members have characterized functions in transferring arabinose and xylose substitutions onto a 1,4-β-xylan backone (Anders et al., 2012). GT75 members are annotated as UDP-Ara mutases (UAM), involved in the conversion of UDP-Arabinopyranose to UDP-Arabinofuranose, which is essential for the generation of the UDP-Ara*f* substrate for arabinoxylan, arabinogalactan protein, and pectic polysaccharide biosynthesis (Hsieh et al., 2015). With the recent finding of arabinoxylan in the papillae produced by barley in response to the attempted penetration of *Blumeria graminis* f.sp. *hordei* (Chowdhury et al., 2014), it is tempting to speculate that GT61 and GT75 family members are broadly implicated in defense responses. GT92 family members play a role in the synthesis of 1,4-β-galactan (Liwanag et al., 2012), which is relatively abundant in tension wood that forms in response to mechanical stress (Andersson-Gunnerås et al., 2006). Therefore, although this broad-brush meta-analysis of CAZy families during abiotic and biotic stress does not take into account differences in individual family member activity, gene family copy number or tissue-specific expression patterns, it identifies a set of CAZy families that are well-characterized in terms of stress response (e.g., GH17, GH18) as well as those that are less well-characterized (GT61, GT75).

**Figure 3:**
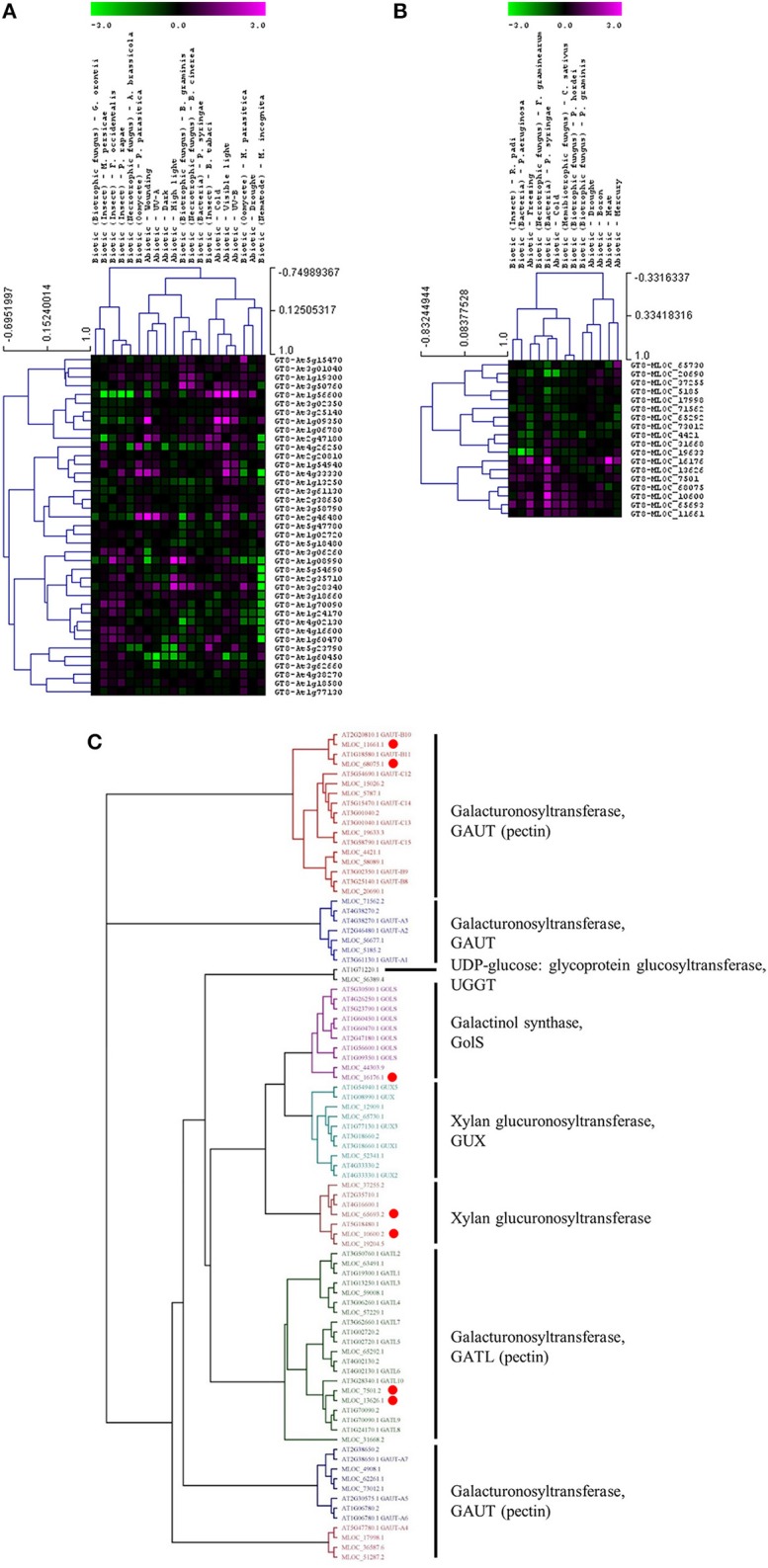
**Analysis of GT8 family members following abiotic and biotic stresses in Arabidopsis (A) and barley (B)**. Transcript abundance was determined through meta-analysis of microarray datasets collected from the Plant Expression Database (PLEXdb; Dash et al., 2012) using the experiments listed in Table 2. Values show the average log(2)-fold induction for representatives of each CAZy gene family present on the Arabidopsis Affymetrix 22K ATH1 genome array and the 22K Barley1 genechip. Hierarchical clustering was performed based on the Pearson correlation coefficients across each dataset and CAZy family. **(C)** Phylogenetic tree of GT8 family members from Arabidopsis and barley with putative functions assigned for each clade. Red dots highlight barley genes that are upregulated in response to stress **(B)**.

**Figure 4:**
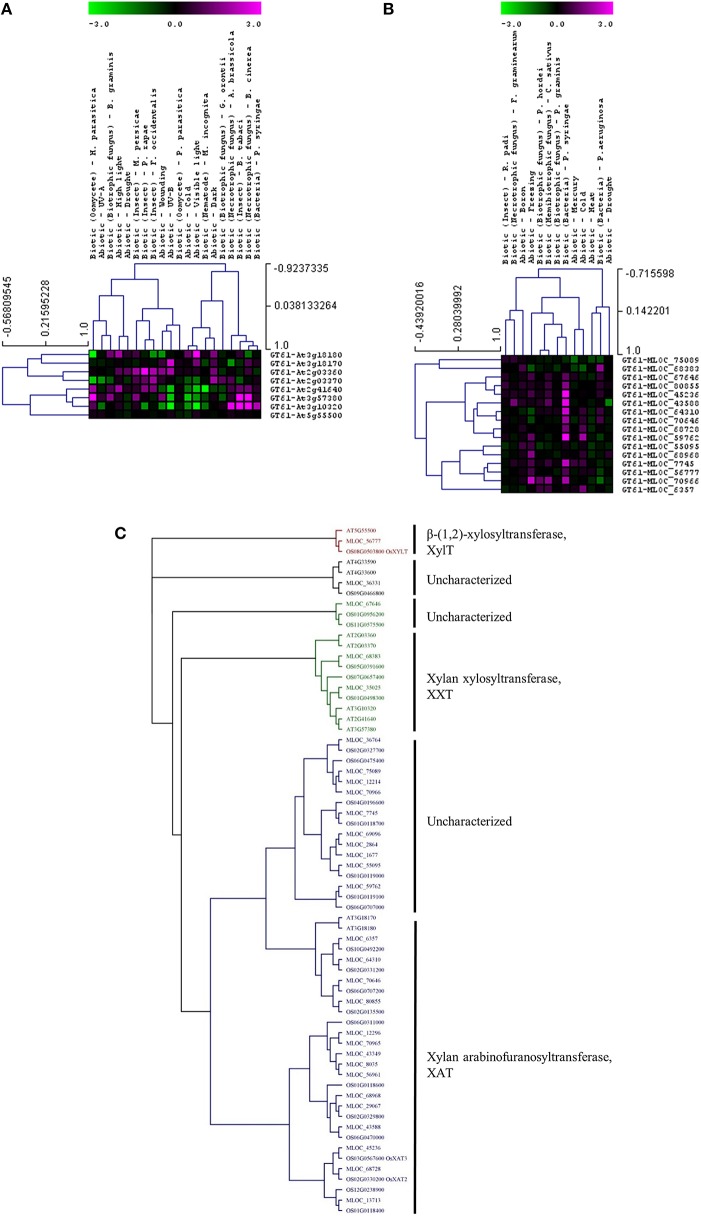
**Analysis of GT61 family members following abiotic and biotic stresses in Arabidopsis (A) and barley (B)**. Transcript abundance was determined through meta-analysis of microarray datasets collected from the Plant Expression Database (PLEXdb; Dash et al., 2012) using the experiments listed in Table 2. Values show the average log(2)-fold induction for representatives of each CAZy gene family present on the Arabidopsis Affymetrix 22K ATH1 genome array and the 22K Barley1 genechip. Hierarchical clustering was performed based on the Pearson correlation coefficients across each dataset and CAZy family. **(C)** Phylogenetic tree of GT61 family members from Arabidopsis, barley, and rice with putative functions assigned for each clade.

Whether members of these CAZy families have specific or similar effects on cell-wall targets can be addressed by characterizing the function of the underlying genes. For example, up-regulated expression of the GT8 and GT61 families highlights a potential role of pectin and xylan synthesis in the plant stress response across both species. However, these families contain members that are involved in many different processes and it is important to assess the expression and function of each gene in more detail. Figure 3 shows the expression levels of each gene from the Arabidopsis (Figure 3A) and the barley (Figure 3B) GT8 gene family. The majority of the Arabidopsis GT8 genes are up-regulated in response to at least one stress, but there appears to be subgroups that respond to specific stresses. Conversely the barley GT8 family is split into two groups, one containing genes that are unchanged or down-regulated in response to stress and the other containing genes which are up-regulated by most of the stresses. Comparison of the stress responsive barley GT8 genes to characterized GT8 family members from Arabidopsis (Figure 3C) suggests that the general stress responsive barley genes are not restricted to clades with a single putative function, but are spread between the galactinol synthase (GolS), xylan glucuronosyltransferase (GUX) and galacturonosyltransferase (GAUT and GATL) activities. Figure 4 shows the expression levels of each gene from the GT61 gene family of Arabidopsis (Figure 4A) and the barley (Figure 4B) GT61 gene family. There is no clear clustering of stress responsive and non-responsive genes as observed for the GT8 family, with GT61 genes upregulated in a number of different stresses for both Arabidopsis and barley. Comparison of the stress responsive barley and Arabidopsis GT61 genes to other GT61 family members that have been functionally characterized (Figure 4C) indicates that the stress responsive genes are not restricted to clades with a single putative function i.e., β-(1,2)-xylosyltransferase (XylT), xylan xylosyltransferase (XXT), and xylan arabinofuranosyltransferase (XAT) activities. Once again this highlights the need for further characterization of cell-wall related genes in stress responses. Conserved changes in CAZy gene expression in different species may indicate that related genes are recruited to act on similar substrates during stress responses. Alternatively, genes from the same family may be recruited to modify different substrates but in a similar way.

## Perspectives and summary

The basis for this review was to consider cell wall and polysaccharide-related activities that influence biotic and abiotic stress responses, and highlight those that might fulfill a common function in promoting remodeling of the cell wall as a direct response to abiotic stress or pathogen attack. Genetic and transgenic evidence suggests that modification of specific cell wall activities has a pronounced effect on stress tolerance. In several cases, similar gene families appear to modulate the effect of distinct biotic and abiotic stresses within and across different species, implying that common mechanisms may have been recruited to target seemingly disparate stress types. This is supported by broader whole-transcriptome analyses, which indicate similar responses of individual cell-wall related genes and even CAZy families to different abiotic and biotic stresses. Whether these overlaps in gene expression lead to similar changes in cell wall structure has yet to be confirmed in most cases, particularly in the case of pectins and xylans which show distinct differences in abundance between monocot and dicot models. Indeed, the functions of many cell wall-related genes have yet to be reported during normal growth and development, let alone during stress responses. This highlights a need to further extend genome editing technologies toward entire CAZy families, and to develop methodologies for chemical cell wall analysis that are high-throughput and capable of being targeted toward single cell-types.

## Author contributions

KH, AL, MT, NS, and JC conceived this review, drafted and revised the manuscript, approved the final version prior to publication and agree to be accountable for all aspects of the work in ensuring that questions related to the accuracy or integrity of any part of the work are appropriately investigated and resolved.

## Funding

KH would like to acknowledge funding from the Scottish Government Research Program. AL, NS and JC were supported by grants from the Australian Research Council. MT was supported by an ARC Future Fellowship.

### Conflict of interest statement

The authors declare that the research was conducted in the absence of any commercial or financial relationships that could be construed as a potential conflict of interest.
